# Acute toxicity of ammonia and nitrite to Siamese fighting fish (*Betta splendens*)

**DOI:** 10.1186/s40850-023-00188-3

**Published:** 2023-11-02

**Authors:** Makiko Kajimura, Kazuyuki Takimoto, Ayaka Takimoto

**Affiliations:** Faculty of Education, Wakayama University, Sakaedani 930, Wakayama, 640-8510 Japan

**Keywords:** Ammonia, Nitrite, *Betta splendens*, LC50, Acute toxicity

## Abstract

The acute toxicity and sublethal effects of ammonia and nitrite on the air-beathing Siamese fighting fish, betta (*Betta splendens*) was studied for 96 h. The LC50 (50% Lethal Concentration) for 96 h for adult bettas to ammonia-N and nitrite-N was 123.4 mM (1.7 g/L, 95% confidence limits: 114.7–130.0 mM) and 24.6 mM (343.6 mg/L, 95% confidence limits: 22.7–26.4 mM) respectively. Exposure to 90 mM ammonia did not affect ammonia and urea excretion rates in bettas. There was no significant difference in values between control and ammonia-loaded (90 mM ammonia) individuals in either brain or liver activities of glutamine synthase, while plasma ammonia levels slightly increased. It appears unlikely that ammonia was converted to urea or amino acids for detoxification. Sublethal nitrite (24.6 mM nitrite) affected plasma nitrite, methemoglobin and hemoglobin. Plasma nitrite values remained much lower than ambient concentrations. Betta has a labyrinth organ and can breathe air. Bettas may temporarily reduce the entry of ammonia and nitrite into the body by increasing the rate of air respiration and reducing the contribution of the gill epithelium, which is highly permeable to these nitrogenous pollutants.

## Introduction

The Siamese fighting fish, betta (*Betta splendens*), native to Thailand, is an ornamental fish with a multicolored body and long, ribbon-like fins. Over the centuries, bettas have been bred for gambling purposes by making two males fight against each other, and to make their appearance more attractive, resulting in a variety of bettas [1, 2]. Today, it is one of the most widely exported ornamental fish in Thailand [2]. In addition to their beauty, their popularity as an ornamental fish is likely due in part to the ease at which they can be kept. Bettas are hardy and can be kept in small aquariums [1]. In fact, they are sold in retail stores in small glasses or glass bottles without filtration or aeration [3]. It has been reported that bettas grow better in 150 ml of water than in 300 ml [4]. In general, fish rearing requires an adequate amount of water. When fish are kept in a small amount of water, the dissolved oxygen content of the water in the container is reduced by the fish’s respiration, resulting in a lack of oxygen. However, bettas have a special organ called the labyrinth organ, a deformed and developed epithelium of the gills, which allows them to breathe air when the amount of dissolved oxygen is low. Dissolved oxygen is not the only reason fish cannot be kept in small containers for long periods of time. Nitrogen compounds in the water, especially ammonia and nitrite (NO_2_^−^), are toxic to fish.

Ammonia is mainly produced when animals break down proteins and amino acids. Ammonia is ionized in water and exists in two forms: un-ionized ammonia (NH_3_) and ammonium ions (NH_4_^+^). In this paper, we distinguish between NH_3_ and NH_4_^+^ and refer to the sum of NH_3_ and NH_4_^+^ as ammonia. The rate at which ammonia is ionized is highly dependent on temperature and pH. The pKa value, which indicates the pH at which half of the ammonia is NH_4_^+^ and half is NH_3_, is about 9–10. Their toxicities differ, with NH_3_ being 300–400 times more toxic compared to NH_4_^+^ [5]. Most teleost fish are ammoniotelic. Ammonia accounts for about 70–90% of total nitrogenous waste, and most of the remainder is excreted in the form of urea, which results from the breakdown of nucleic acids and arginine [6]. Fish excrete ammonia into the water by diffusion through their gills mainly in the form of NH_3_, and they keep ammonia levels in the body low [7–10]. However, when kept in small containers, the ammonia they excrete accumulates in high concentrations in the containers. In addition, ammonia is also produced from the decomposition of feces and leftover food. As the concentration of ammonia in the water increases, NH_3_ flows into the body and accumulates due to an inverse diffusion gradient [11], in some cases to lethal levels. However, some fish are known to synthesize urea and excrete it from the body under conditions that restrict the excretion of ammonia, such as in air or under ammonia exposure, by converting ammonia into glutamine through the action of glutamine synthetase and introducing it into the urea cycle [12–15].

In addition, through the nitrification process, ammonia in the water is oxidized to nitrite and then to nitric acid. Nitrite is known to be highly toxic to fish. Nitrite that enters the body oxidizes the hemoglobin in red blood cells, converting it to methemoglobin, which has no oxygen-carrying capacity, leading to hypoxia [16–19]. Normally, nitrite concentrations are low, but in closed environments such as aquaculture, the nitrification process can be imbalanced and nitrite concentrations can increase.

Although rearing bettas in a closed environment without filtration equipment and in small amounts of water is likely to increase the concentrations of ammonia and nitrite in the water, there have been no reports on the tolerance to these substances in bettas. The objective of this study was to determine if bettas have a high tolerance to ammonia and nitrite.

## Materials and methods

### Fish

Male *Betta splendens* (1.1–2.4 g) were obtained from a local pet store in Wakayama Prefecture, Japan. The fish were held individually in 1000 mL glass beakers with 600 mL of aerated Wakayama tap water (hardness 65 mg/L as CaCO_3_, pH 6.8, [Ca_2_^+^] 21.5 mg/L, [Mg_2_^+^] 2.7 mg/L, [Na^+^] 9.7 mg/L, [Cl^–^] 11.1 mg/L) at 25 ± 0.5°C in for at least two weeks prior to experimentation under a constant photoperiod of 12:12 L:D. Water was changed twice a week. Fish were supplied a commercial diet (Hikari betta, 38% protein, Kyorin, Japan) once every day to satiation. Food was withheld for 2 days before and during the experiments. Because bettas are air-breathing fish, no aeration was used during the acclimation and experiment period. White paper partitions were placed between the containers so that no other fish could be seen.

### LC50 for ammonia and nitrite

Fish (n = 144) were subjected to different concentrations of ammonia or nitrite for 96 h to determine the lethal concentrations (LC50). For each treatment, eight fish were placed in clean containers individually with 600 mL test water under the constant photoperiod. A clear plastic sheet was placed over the container to prevent evaporation during processing.

For ammonia tolerance, fish were exposed to nine concentrations of NH_4_Cl: 60, 80, 90, 100, 110, 120, 130, 140, and 150 mM. In the ammonia tolerance experiment, water and containers were changed every 24 h by gently transferring fish with a fish net to prevent significant decrease in ammonia concentration due to volatilization. For nitrite tolerance, fish were exposed to nine concentrations of NaNO_2_: 10, 15, 20, 22, 24, 25, 26, 28, and 30 mM. During the experiment, the same water in the container was used for 96 h.

Fish were monitored for mortality at least 3 times daily. Water samples (3 mL) were withdrawn with a pipette to determine the concentrations of ammonia/nitrite. All water samples were frozen at -20°C until analysis. The fish were weighed after the experiment.

### Ammonia exposure on nitrogen excretion, plasma ammonia, glutamine synthetase

In the present study, ammonia was measured as total ammonia (NH_3_ + NH_4_^+^). The corresponding fractions of un-ionized ammonia (NH_3_) and ammonium (NH_4_^+^) were calculated in accordance with the Henderson–Hasselbalch equation:

$${\rm{pH = pKAmm}}\,{\rm{ + }}\,{\rm{log[N}}{{\rm{H}}_3}{\rm{]/[N}}{{\rm{H}}_4}^ + {\rm{]}}$$

where pKAmm is based on temperature [20].

Fish were placed individually in the containers with 400 mL of fresh water (FW) or 90 mM ammonia dissolved in FW for 96 h. Water pH was measured at 0, 24, 48, 72, and 96 h. External water was collected and frozen for ammonia and urea analysis at 0, 24, 48, 72, and 96 h. After measuring pH and taking water samples, the external water and the containers were changed daily. After 96 h exposure, the fish were anesthetized by immersion in tricaine methanesulphonate (MS-222; 200 mg/L), rinsed with clean water, dried with a clean paper towel to removed water on the body surface, and weighed. Blood samples were collected by tail ablation with a scalpel and hematocrit tubes, then transferred into centrifuge tubes and plasma samples were obtained by centrifugation at 9000 g for 2 min. The liver and brain were removed. The plasma and tissues were immediately frozen in liquid nitrogen and stored at -80°C until further analyses.

Ammonia in water samples was determined by the indophenol blue method [21]. Plasma ammonia was measured enzymatically on the first thaw of nondeproteinized plasma using glutamate dehydrogenase (Sigma-Aldrich, ammonia assay kit, product AA0100). Urea-N concentrations in water and plasma were measured using the diacetyl-monoxime method [22].

For determination of activity of glutamine synthetase (GS; EC 6.3.1.2), tissues were homogenized on ice in 20–50 volumes of homogenization buffer (20 mM K_2_HPO_4_, 10 mM HEPES, 0.5 mM EDTA, 1 mM dithiothreitol, 50% glycerol adjusted with NaOH to pH 7.5 at 24°C) using a BioMasher (Nippi,Tokyo, Japan). Homogenates were centrifuged at 8,000 g for 20 min at 4°C. The supernatant was used for assaying the activity of GS by previously described methods [23–25].

### Nitrite exposure on plasma nitrite, methemoglobin, hematocrit, hemoglobin level

Fish were placed individually in containers with 600 mL of FW or 24.6 mM NaNO_2_ dissolved in FW for 96 h. After 96 h exposure, the fish were anesthetized by immersion in MS-222 (200 mg/L), rinsed with clean water, dried with paper towel and weighed. Blood samples were collected by tail ablation with a scalpel and hematocrit tubes. Hematological analyses were performed immediately after blood sampling. For plasma nitrite, whole blood was transferred into centrifuge tubes and plasma samples were obtained by centrifugation at 9000 g for 2 min and stored at -80°C until further analyses.

For hematological analyses, hematocrit (Hct) and hemoglobin (Hb) concentrations were determined using whole blood. The Hct value was determined using a capillary hematocrit tube and hematocrit centrifuge (25°C, 14,000 g for 2 min). The Hb concentration was measured using the cyanmethemoglobin technique by Drabkin’s reagent (Sigma-Aldrich, D5941). Methemglobin (MetHb) in the blood of fish was measured using the Evelyn and Malloy method [26, 27]. Plasma nitrite was measured with a modified naphthylethylenediamine method [28].

### Statistical analysis

All data are given as means ± S.E. (n = number of animals). Probit analysis was used to evaluate LC50 (SPSS. IBM 25). An independent t-test was used to determine significance. For all analyses, P < 0.05 was considered significant.

## Results

### Lethal concentration tests for ammonia and nitrite

There was no mortality for fish in concentrations of nitrite up to 20 mM and ammonia up to 90 mM during 96 h of the experiment. The 96 h LC50 value of nitrite-N was 24.6 mM (343.6 mg/L) (Table 1). The 96 h LC50 values of total ammonia-N was 123.4 mM (1.7 g /L) and NH_3_-N was 551.2 µM (7.7 mg/L).

**Table 1:** Calculated LC50 values (mM) of ammonia (NH_3_ + NH_4_^+^), unionized ammonia (NH_3_), and nitrite for *Betta splendens* with 95% confidence limits (in parenthesis) for various exposure times

Time (hour)	Ammonia (NH_3_ + NH_4_^+^) (mM)	Unionized ammonia (NH_3_) (mM)	Nitrite (mM)
24	129.70 (121.62–140.7)	0.58 (0.54–0.63)	28.97 (27.00-35.31)
48	125.65 (117.79-135.03)	0.56 (0.53–0.60)	28.03 (26.05–33.12)
72	125.65 (117.79-135.03)	0.56 (0.53–0.60)	27.37 (25.48–31.41)
96	123.4 (114.69-130.03)	0.55 (0.51–0.58)	24.57 (22.69–26.42)

### Nitrogen excretion

**Fig. 1:**
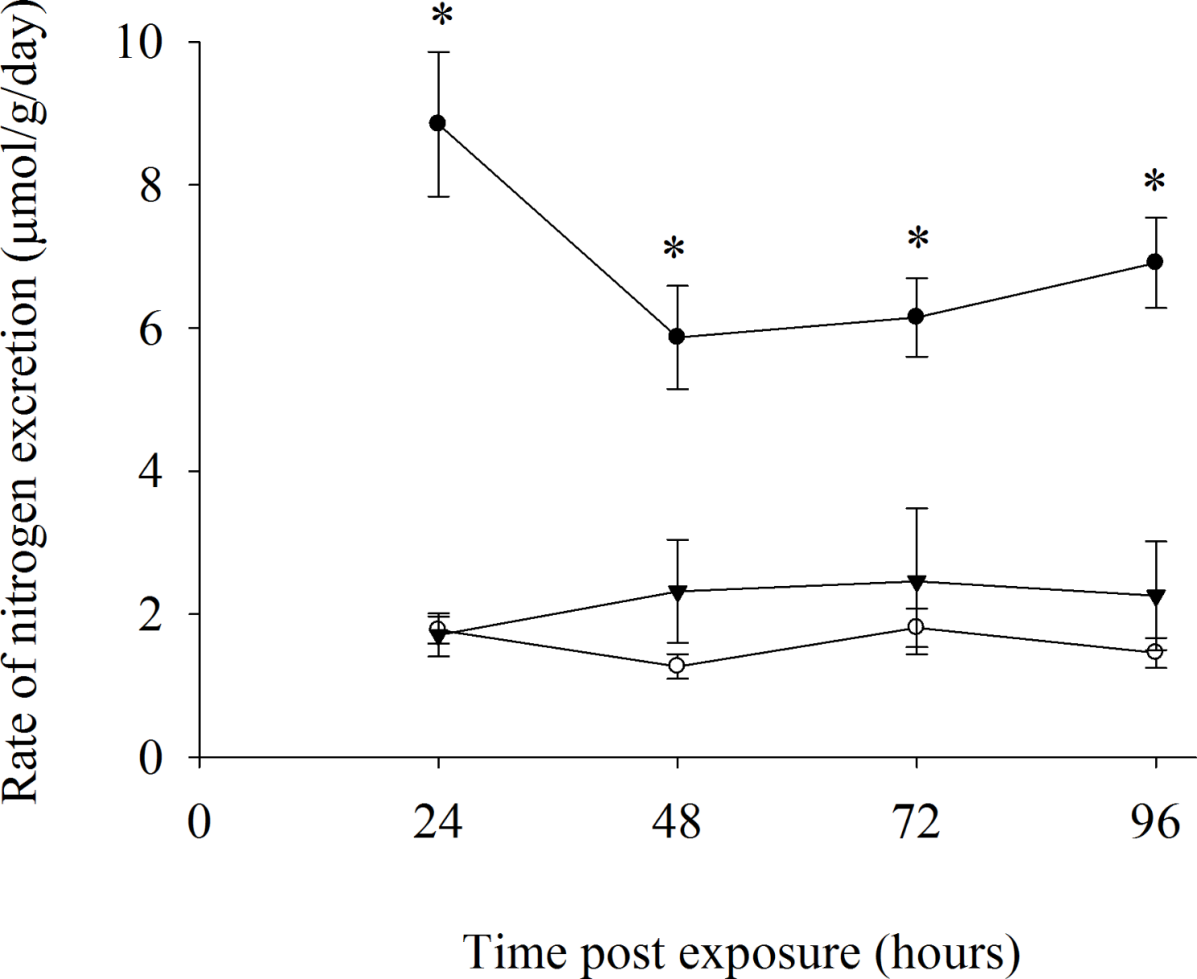
Changes in ammonia and urea excretion rates over time in *Betta splendens* exposed to 90 mM ammonia (urea excretion: filled inverted triangle, n = 5) and in the control fish (urea excretion: open circles; ammonia excretion: filled circles, n = 6). Values are means ± standard error. An asterisk indicates a significant difference (*P* < 0.05) from the corresponding control urea excretion value

The control fish excreted nitrogen waste mostly as ammonia, with urea excretion accounting for less than 23% of total nitrogen excretion (ammonia-N + urea-N) (Fig. 1). Bettas are therefore ammoniotelic. Although ammonia excretion dropped between 24 and 48 h significantly, the rates of ammonia and urea excretion were rather stable. The urea excretion rate of 90 mM ammonia exposed fish was similar to that of the control fish over 96 h and showed no significant variation.

### Effects of ammonia exposure

Plasma ammonia levels and brain and liver glutamine synthase (GS) activity levels in control and ammonia-loaded individuals are shown in Table 2. Exposure to 90 mM ammonia significantly increased plasma ammonia. There was no significant difference in activity values between control and ammonia-loaded individuals in either brain or liver for activities of glutamine synthase.

**Table 2:** Mean and standard error (SE) values for plasma ammonia concentration and glutamine synthetase in liver and brain in *Betta splendens* exposed to sublethal ammonia concentrations (90 mM) for 96 h. Asterisks indicate a significant difference from the control value: *P < 0.05

	Plasma ammonia (mM)		GS activity (µmol/min/g)			
		n	Liver	n	Brain	n
Control	0.69 (0.13)	7	1.74 (0.28)	8	34.66 (2.31)	8
90 mM ammonia	1.18 (0.15)*	7	1.08 (0.20)	7	33.23 (2.45)	8

### Effect of nitrite exposure

As shown in Table 3, nitrite concentration in plasma and methemoglobin value of nitrite-exposed fish was 39 times and 64 times those of controls, respectively. Hemoglobin levels in fish exposed to nitrite were higher than controls, but hematocrit levels were unchanged.

**Table 3:** Mean and standard error (SE) values for plasma nitrite concentration, methemoglobin, hemoglobin, and hematocrit in *Betta splendens* exposed to sublethal nitrite concentrations (24.4 mM) for 96 h. Asterisks indicate a significant difference from the control value: *** *P* < 0.001

	Plasma nitrite		Methemoglobin		Hemoglobin		Hematocrit	
	µM	n	%	n	g/dL	n	%	n
Control	33.9 (3.2)	8	0.8 (0.6)	11	12.8 (1.0)	11	33.1 (2.0)	11
24.4 mM nitrite	1357.1 (221.9)***	7	51.5 (6.6)***	11	18.6 (1.2)***	11	35.1 (2.3)	11

## Discussion

The 96 h LC50 for bettas for ammonia-N and NH_3_-N was 123.4 mM (1.7 g/L) and 551.2 µM (7.7 mg /L), respectively. The 96 h LC50 for freshwater fish is generally in the range of 0.068-2.0 mg/L NH_3_ [29], indicating that bettas have a very high tolerance to ammonia. Comparisons of previous studies have reported that air-breathing fish are generally more tolerant to ammonia than water-breathing fish [30]. This is because air-breathing fish need to temporarily deal with the endogenous ammonia that accumulates in their bodies while water is not available. The mudskipper *Periophthalmodon schlosseri*, showed a 96-h LC50 of 536 µM for NH_3_ [31]. The air-breathing catfish *Heteropneustes fossilis* was also reported to be able to survive at 75 mM NH_4_Cl (pH 7.2) for 4 weeks [32].

Bettas accumulated ammonia under ammonia-loaded conditions, with the plasma ammonia content of bettas increasing 1.7-fold under ammonia exposure. On the other hand, urea excretion did not change, indicating that bettas do not appear to convert ammonia into urea for emission in short periods such as 96 h. The activity of glutamine synthase was also unchanged in this study, and it is unlikely that ammonia is actively converted to glutamine for detoxification under acute ammonia loading. This is similar to two other air-breathing fish, the swamp eel *Monopterus albus* and the weather loach *Misgurnus anguillicaudatus*, which may accumulate high concentrations of endogenous ammonia in the body, such as in the muscle and liver, and in plasma during exposure to air, when ammonia excretion is limited [33, 34].

The 96 h LC50 of nitrite-N for bettas was 24.6 mM (343.6 mg /L). The sensitivity of fish to nitrite also varies widely among species, and its toxicity is known to be affected by a variety of factors, including exposure time, fish size, ionic composition in the water, and temperature [18, 35, 36]. In particular, because nitrite is taken up across the chloride cells in gills in competition with chloride, the chloride concentration in the water strongly affects nitrite sensitivity, with higher chloride ion concentrations resulting in lower toxicity [16–18, 37]. In low chloride ion concentrations (< 30 mg/L, 0.845 mM) like those used in the present study, the 96 h LC50 for most fish is tens of mg NO_2_^−^ /L [19], however highly tolerant fish have been reported, such as juvenile Guadalupe bass (*Micropterus treculi*) 187.6 mg NO_2_-N/L [38], Green sunfish (*Lepomis cyanellus*) 526.8 mg NO_2_^−^/L (160.1 mg NO_2_-N/L) [39], and Largemouth bass (*Micropteus salmoides*) 140.2 mg NO_2_-N/L [40]. Bayley et al. (2020) [30] have noted that air-breathing fish may be highly tolerant not only to nitrite but also ammonia, with LC50 values of 7.82 mM for the clown knifefish (*Chitala ornata*) [41] and 31 mM for the swamp eel (*Monopterus albus*) [30].

When exposed to nitrite, freshwater fish take up nitrite mainly from gill chloride cells and may accumulate high concentrations in plasma [18]. In some freshwater fish, plasma nitrite concentrations have been reported to reach several to dozens of times the ambient concentrations [17, 40, 42, 43]. The concentration of plasma nitrite is considered a major determinant of nitrite tolerance in fish, with a negative correlation [39]. The fact that bettas with high nitrite tolerance do not accumulate high levels of nitrite in their plasma is consistent with this suggestion. Differences in nitrite tolerance among species of freshwater fish are closely related to differences in chloride absorption capacity in the gills [44]. This suggests that the chloride absorption capacity of gills may be low in bettas.

Nitrite entering the bloodstream renders the divalent iron ions in hemoglobin unable to transport oxygen, as they are oxidized by nitrite to trivalent iron ions [17, 19, 37]. Fish usually do not die if methemoglobin concentrations are below 50% [16], and up to 70% appears to be tolerated without significant mortality [37]. Methemoglobin levels in bettas remained relatively low at 51.5% under semi-lethal concentrations of nitrite. In addition, although decreases in hemoglobin and hematocrit are commonly observed in fish exposed to nitrite [17, 37], hemoglobin concentrations increased slightly and hematocrit values were unchanged in the present study. In bettas, nitrite does not appear to have a significant effect on these blood parameters. This may be because plasma nitrite levels are kept low.

Under ammonia and nitrite exposure for 96 h, although ammonia and nitrite in plasma in bettas increased significantly, the values were kept much lower than ambient concentrations. Betta has a labyrinth organ and can breathe air. Bettas might temporarily reduce the entry of ammonia and nitrite into the body by reducing the contribution of the gill epithelium, which is highly permeable to ammonia and nitrite, and by increasing the proportion of air respiration. This may be why they can tolerate extremely high concentrations.

In nature, ammonia and nitrite in water are usually converted by nitrification by microorganisms and rarely accumulate in high concentrations. How did bettas develop this high tolerance? Bettas are highly territorial, and if males are kept together in a small tank, they will fight violently, so they must be kept separately. In Thailand, where betta breeding is popular, bettas are often kept individually in half-pint liquor bottles with a cut at the shoulder to allow for water drainage, water changes are done directly into the bottles, and they are given food and raised in these bottles until shipment [45]. They are also placed individually in small bags containing about 90% air and a small amount of water during shipment, and airlifted all over the world [45]. It is possible that under such an aquaculture environment or during transportation, bettas were exposed to high concentrations of ammonia and nitrite due to decomposition of excreta and food, and that selection may have occurred with the more tolerant bettas surviving. In order to determine the reason for the high tolerance in bettas, environmental studies of the habitat of wild types and a comparison to bred types would be needed.

## Data Availability

Not applicable.

## References

[1] Smith HM (1945). The fresh-water fishes of Siam, or Thailand. Bull United States Natl Museum.

[2] Sermwatanakul A (2019). Capacitating the local farmers to enhance global marketing of Thailand’s national aquatic animal, the siamese fighting fish. Fish for the People.

[3] Pleeging CCF, Moons CPH (2017). Potential welfare issues of the siamese fighting fish (*Betta splendens*) at the retailer and in the hobbyist aquarium. Vlaams Diergen Tijds.

[4] Saekhow S, Thongprajukaew K, Phromkunthong W, Sae-khoo H (2018). Minimal water volume for intensively producing male siamese fighting fish (*Betta splendens* Regan, 1910). Fish Physiol Biochem.

[5] Haywood GP (1983). Ammonia toxicity in teleost fish: a review. Can Tech Rep Fish Aquat Sci.

[6] Wood CM, Evans DH (1993). Ammonia and urea metabolism and excretion. The physiology of fishes.

[7] Wilkie MP (1997). Mechanisms of ammonia excretion across fish gills. Comp Biochem Physiol A Physiol.

[8] Wilkie MP (2002). Ammonia excretion and urea handling by fish gills: present understanding and future research challenges. J Exp Zool.

[9] Evans DH, Piermarini PM, Choe KP (2005). The multifunctional fish gill: dominant site of gas exchange, osmorgulation, acid-base regulation and excretion of nitrogenous waste. Physiol Rev.

[10] Wright PA, Wood CM (2009). A new paradigm for ammonia excretion in aquatic animals: role of Rhesus (Rh) glycoproteins. J Exp Biol.

[11] Gilmour KM (2022). Pushing back against high environmental ammonia levels: a model for active NH_4_^+^ excretion. Acta Physiol (Oxf).

[12] Randall DJ, Wood CM, Perry SF, Bergman H, Maloiy GMO, Mommsen TP, Wright PA (1989). Urea excretion as a strategy for survival in a fish living in a very alkaline environment. Nature.

[13] Walsh PJ, Danulat E, Mommsen TP (1990). Variation in urea excretion in the gulf toadfish *Opsanus beta*. Mar Biol.

[14] Iwata K, Kajimura M, Sakamoto T (2000). Functional ureogenesis in the gobiid fish, *Mugilogobius abei*. J Exp Biol.

[15] Ip YK, Chew SF (2018). Air-breathing and excretory nitrogen metabolism in fishes. Acta Histochem.

[16] Lewis WM, Morris DP (1986). Toxicity of nitrite to fish: a review. Trans Am Fish Soc.

[17] Jensen FB (2003). Nitrite disrupts multiple physiological functions in aquatic animals. Comp Biochem Physiol Part A Mol Integr Physiol.

[18] Kroupova H, Machova J, Svobodova Z (2005). Nitrite influence on fish: a review. Vet Med.

[19] Kroupova HK, Valentova O, Svobodova Z, Sauer P, Machova J (2018). Toxic effects of nitrite on freshwater organisms: a review. Rev Aquac.

[20] Cameron JN, Heisler N (1983). Studies of ammonia in the rainbow trout: physicochemical parameters, acid-base behaviour and respiratory clearance. J Exp Biol.

[21] Ivancic L, Degobbis D (1984). An optimal manual procedure for ammonia analysis in natural water by the indophenol blue method. Water Res.

[22] Rahmatullah M, Boyde TR (1980). Improvements in the determination of urea using diacetyl monoxime: methods with and without deproteinization. Clin Chim Acta.

[23] Mommsen TP, Walsh PJ (1989). Evolution of urea synthesis in vertebrates: the piscine connection. Science.

[24] Barber ML, Walsh PJ (1993). Interactions of acid-base status and nitrogen excretion and metabolism in the ureogenic teleost, *Opsanus beta*. J Exp Biol.

[25] Walsh PJ, Tucker B, Hopkins T (1994). Effects of confinement/crowding on ureogenesis in the gulf toadfish *Opsanus beta*. J Exp Biol.

[26] Evelyn KA, Malloy HAT (1938). Microdetermination of oxyhemoglobin, methemoglobin and sulfhemoglobin in a single sample of blood. J Biol Chem.

[27] Lacey JA, Rodnick KJ (2002). Important considerations for methaemoglobin measurement in fish blood: assay choice and storage conditions. J Fish Biol.

[28] Strickland JDH, Parsons TR (1968). Determination of reactive nitrite. Fisheries Res Board Can.

[29] Eddy FB (2005). Ammonia in estuaries and effects on fish. J Fish Biol.

[30] Bayley M, Damsgaard C, Cong N, Phuong N, Do H, Farrell A, Brauner C, Benfey T (2020). Aquaculture of air-breathing fishes. Fish Physiology.

[31] Peng KW, Chew SF, Lim CB, Kuah SSL, Kok WK, Ip YK (1998). The mudskippers *Periophthalmodon schlosseri* and *Boleophthalmus boddaerti* can tolerate environmental NH_3_ concentrations of 446 and 36 µm, respectively. Fish Physiol Biochem.

[32] Saha N, Ratha BK (1994). Induction of ornithine–urea cycle in a freshwater teleost, *Heteropneustes fossilis*, exposed to high concentrations of ammonium chloride. Comp Biochem Physiol B Comp Biochem.

[33] Chew SF, Jin Y, Ip YK (2001). The loach *Misgurnus anguillicaudatus* reduces amino acid catabolism and accumulates alanine and glutamine during aerial exposure. Physiol Biochem Zool.

[34] Tay SLA, Chew SF, Ip YK (2003). The swamp eel *Monopterus albus* reduces endogenous ammonia production and detoxifies ammonia to glutamine during aerial exposure. J Exp Biol.

[35] Palachek RM, Tomasso JR (1984). Nitrite toxicity to fathead minnows: effect of fish weight. Bull Environ Contam Toxicol.

[36] Atwood HL, Fontenot QC, Tomasso JR, Isely JJ (2001). Toxicity of nitrite to Nile tilapia: effect of fish size and environmental chloride. N Am J Aquac.

[37] Ciji A, Akhtar MS (2020). Nitrite implications and its management strategies in aquaculture: a review. Rev Aquac.

[38] Tomasso JR, Carmichael GJ (1986). Acute toxicity of ammonia, nitrite, and nitrate to the Guadalupe bass, *Micropterus treculi*. Bull Environ Contam Toxicol.

[39] Tomasso JR (1986). Comparative toxicity of nitrite to freshwater fishes. Aquat Toxicol.

[40] Palachek RM, Tomasso JR (1984). Toxicity of nitrite to channel catfish (*Ictalurus punctatus*), tilapia (*Tilapia aurea*), and largemouth bass (*Micropterus salmoides*): evidence for a nitrite exclusion mechanism. Can J Fish Aquat Sci.

[41] Gam LTH, Jensen FB, Damsgaard C, Huong DTT, Phuong NT, Bayley M (2017). Extreme Nitrite tolerance in the clown knifefish *Chitala ornata* is linked to up-regulation of methaemoglobin reductase activity. Aquat Toxicol.

[42] Margiocco C, Arillo A, Mensi P, Schenone G (1983). Nitrite bioaccumulation in *Salmo Gairdneri* rich. And hematological consequences. Aquat Toxicol.

[43] Roques JAC, Schram E, Spanings T, van Schaik T, Abbink W, Boerrigter J, de Vries P, van de Vis H, Flik G (2015). The impact of elevated water nitrite concentration on physiology, growth and feed intake of African catfish *Clarias gariepinus* (Burchell 1822). Aquac Res.

[44] Tomasso JR, Grosell M (2005). Physiological basis for large differences in resistance to nitrite among freshwater and freshwater-acclimated euryhaline fishes. Environ Sci Technol.

[45] Watson C, DiMaggio M, Hill J, Tuckett Q, Yanong R (2019). Evolution, culture, and care for Betta splendens. EDIS.

